# Exploring how sensory dominance modulated by modality-specific expectation: an event-related potential study

**DOI:** 10.3389/fpsyg.2025.1548100

**Published:** 2025-02-17

**Authors:** Xiaoyu Tang, Dandan Fan, Xueli Wang, Zepeng Xing, Shilong Yu, Aijun Wang, Hongtao Yu

**Affiliations:** ^1^School of Psychology, Liaoning Collaborative Innovation Center of Children and Adolescents Healthy Personality Assessment and Cultivation, Liaoning Normal University, Dalian, China; ^2^College of Humanities and Social Sciences, Yunnan Agricultural University, Kunming, China; ^3^Center for Magnetic Resonance Imaging Research & Key Laboratory of Brain-Machine Intelligence for Information Behavior (Ministry of Education and Shanghai), School of Business and Management, Shanghai International Studies University, Shanghai, China; ^4^Department of Psychology, Soochow University, Suzhou, China

**Keywords:** Colavita effect, modality expectation, sensory dominance, response precedence, event-related potentials

## Abstract

The Colavita visual dominance effect refers to the phenomenon in which tend to respond only or preferentially to visual stimuli of bimodal audiovisual stimulus. Previous evidence has indicated that sensory dominance can be modulated by top-down expectation. However, it remains unclear how expectations directed toward a single sensory modality influence Colavita visual dominance at the electrophysiology level. Using event-related potential (ERP) measurements, we investigated how modality expectation modulates sensory dominance by manipulating the different unimodal target probabilities used in previous related Colavita studies. For the behavioral results, a significantly larger visual dominance effect was found when the modality expectation was directed to the visual sensory condition (40% V:10% A). Further ERPs results revealed that the mean amplitude of P2 (200–250 ms) in the central-parietal region was larger in the visual precedence auditory response (V_A) type than in the auditory precedence visual response (A_V) type when modality expectation was directed to visual sensory stimuli (40% V:10% A). In contrast, the mean amplitude of N2 (290–330 ms) in the frontal region was larger for the V_A type than in the A_V type when modality expectation was directed to the auditory sensory stimuli (10% V:40% A). Additionally, for the A_V type N1 (150–170 ms) in the frontal region was larger in visual versus auditory expectation condition. Overall, the study tentatively suggested that increasing unimodal target probability may lead to greater top-down expectation direct to target modality stimulus, and then sensory dominance emerges in the late phase when participant response to visual stimuli of bimodal audiovisual stimulus.

## 1 Introduction

To successfully perceive an external environment event, the human brain must receive signals from multiple sensory systems and then integrate these signals as a unified whole (Stein and Stanford, 2008; Lewkowicz and Ghazanfar, 2009; Talsma et al., 2010; Xi et al., 2023). Multisensory studies have widely reported that the brain does not give equal weight to signals simultaneously presented in different sensory modalities considering the fact that limited cognitive resources must be allocated to those signals from modalities with higher process priority (Witten and Knudsen, 2005; Manns and Güntürkün, 2009; Ghazanfar and Lemus, 2010). This means that information from one sensory modality is preferentially processed and eventually dominates behavior and awareness; this phenomenon is referred to as the sensory dominance effect (Hecht and Reiner, 2009; Murray et al., 2018). A classical example of sensory dominance is the Colavita visual dominance effect, in which visual information is preferentially processed and eventually dominates other sensory modalities (Colavita, 1974; Hartcher-O'Brien et al., 2008; Koppen et al., 2009).

Colavita described an experiment in which participants were asked to press one button for responding to unimodal visual stimuli (e.g., an incandescent light) and another button for responding to unimodal auditory stimuli (e.g., an SPL tone) (Colavita, 1974). The study revealed that participants tended to respond only or preferentially to visual stimuli when the visual and auditory stimuli were delivered simultaneously (Colavita, 1974). In particular, some studies have reported that the visual dominance magnitude of bimodal audiovisual trials with preceding visual responses and delayed auditory responses (termed V_A) is significantly larger than that of preceding auditory responses and delayed visual response trials (termed A_V) (Li et al., 2017; Fang et al., 2020; Wang et al., 2021). Additionally, event-related potential (ERP) evidence revealed that V_A trials elicited a significantly more positive amplitude than those A_V trials, particularly over the centroparietal regions, during the later post-perceptual phases between 250 and 400 ms after stimulus onset (Huang et al., 2015). An early attention hypothesis suggested that visual stimuli are essentially less alerting than are stimuli in other sensory modalities (e.g., auditory) (Posner et al., 1976). Hence, to compensate for the low alertness of visual stimuli, participant’s attention is deliberately biased toward vision, giving rise to a visual dominance effect during the later phases (Posner et al., 1976; Chen and Huang, 2021). This finding was supported by previous Colavita studies, which also indicated that the visual dominance could be modulated by top-down factors (Posner et al., 1976; Langner et al., 2011; Chen and Huang, 2021).

Expectation refers to brain states that reflect prior information about what is possible or probable in the forthcoming sensory environment (Summerfield and Egner, 2009). Expectation may influence the direction of top-down endogenous attention distribution so that individuals focus more attention on upcoming information that matches expectations, and endogenous attention can affect subsequent expectations (Kok et al., 2012; Summerfield and de Lange, 2014; Dugué et al., 2020). This interaction process is widely suggested as a dynamic and continuous regulatory mechanism (Summerfield and Egner, 2009; Summerfield and de Lange, 2014; Rungratsameetaweemana and Serences, 2019). Studies have suggested that when expectations are directed to one special modality, they can influence how the brain processes sensory information, resulting in prioritized processing or heightened responses to specific sensory modality (Langner et al., 2011; Hutmacher, 2019; Zuanazzi and Noppeney, 2020). Previous Colavita visual dominance studies reported that one possible method for manipulating modality expectation was changing the relative probability of occurrence of unimodal auditory and visual targets (Sinnett et al., 2007; Wang et al., 2023). For example, Sinnett et al. (2007) investigated the modulatory effect of modality expectation on the Colavita visual dominance effect by varying the probability of unimodal visual and auditory stimuli. The author suggested that expectation bias toward visual modality occurred when the probability of occurrence of visual target increased when the probability of visual, auditory, and audiovisual targets is 3:1:1, thereby causing the Colavita visual dominance effect. Notably, when the probability of visual, auditory, and audiovisual targets is 1:3:1, expectation bias toward the auditory modality, the Colavita visual dominance effect does not emerge or reverse (Egeth and Sager, 1977; Zampini et al., 2005; Sinnett et al., 2007; Koppen and Spence, 2007a, 2007b). In our previous study, we adjusted the probability of unimodal stimuli to 10% V:40% A, 25% V:25% A, and 40% V:10% A, and found that the setting of this probability of unimodal stimuli can effectively test the influence of expectation on the Colavita visual dominance effect (Wang et al., 2023). In particular, few studies have investigated the electrophysiology mechanism under the Colavita visual dominance effect via ERP measurements (Stekelenburg and Keetels, 2016; Li et al., 2017). These studies have focused mainly on the effects of synesthetic congruency (Stekelenburg and Keetels, 2016) or the lateralized readiness potential of the visual dominance effect (Li et al., 2017). Early top-down expectation modulation of N1 (130–150 ms) component in the frontal and occipital regions has been found in some studies of the expectation effect on object processing (Stojanoski and Niemeier, 2015), and other studies have shown that expectations alter ERP around P2 (180–270 ms) component in the occipitoparietal regions or N2 (312–340 ms) component in the frontal regions (Melloni et al., 2011; Stojanoski and Niemeier, 2011; Stojanoski and Niemeier, 2014, 2015). However, until now, it has remained unclear how these ERP components involved in sensory dominance with modality expectation.

The present study aimed to investigate the modulatory effect of modality expectation on the visual dominance by manipulating the unimodal target probability, based on the previous related studies (Zampini et al., 2005; Sinnett et al., 2007) as well as our previous findings (Wang et al., 2021). The present study modulated the probability of unimodal visual and auditory stimuli to 10% visual:40% auditory, 25% visual:25% auditory and 40% visual:10% auditory, whereas the percentage of bimodal audiovisual stimulus was 50%. Biased competition theory assumes that audiovisual sensory systems compete with each other when audiovisual information reaches the brain, neural representations that dominate sensory modality may suppress neural representations in the other modality (Desimone and Duncan, 1995; Duncan et al., 1997; Spence et al., 2012). Thus, the study tentatively hypothesized that the Colavita visual dominance effect occurs when expectations favor visual modality stimuli. We also expected that the effect of modality expectation on sensory dominance would be related to some ERP components. Specifically, we predicted that (a) in the A_V type, the N1 amplitude would be larger in visual expectation condition compared to auditory expectation condition; (b) in visual expectation condition, the P2 amplitude in V_A type would be significantly larger than in A_V type; and (c) in auditory expectation condition, the N2 amplitude in V_A type would be significantly larger than in A_V type.

## 2 Materials and methods

### 2.1 Participants

The study requires a minimum of 17 participants based on the G*Power toolbox calculations (Faul et al., 2007). Thirty participants were recruited recruitment advertisements to participate in the experiment. Four participants were excluded due to poor performance, leaving 26 participants in the final analysis (mean age: 23.4 ± 1.8 years; 13 females), all of whom were right-handed. All participants had normal vision and hearing and no neurological or psychiatric disorders. At the end of the experiment, each participant was given 100 RMB as a reward. Prior to the experiment, all participants provided their consent by completing a consent form, which was approved by the Ethics Committee of Liaoning Normal University and was provided by the principles expressed in the Declaration of Helsinki.

### 2.2 Stimuli and procedure

**Figure 1:**
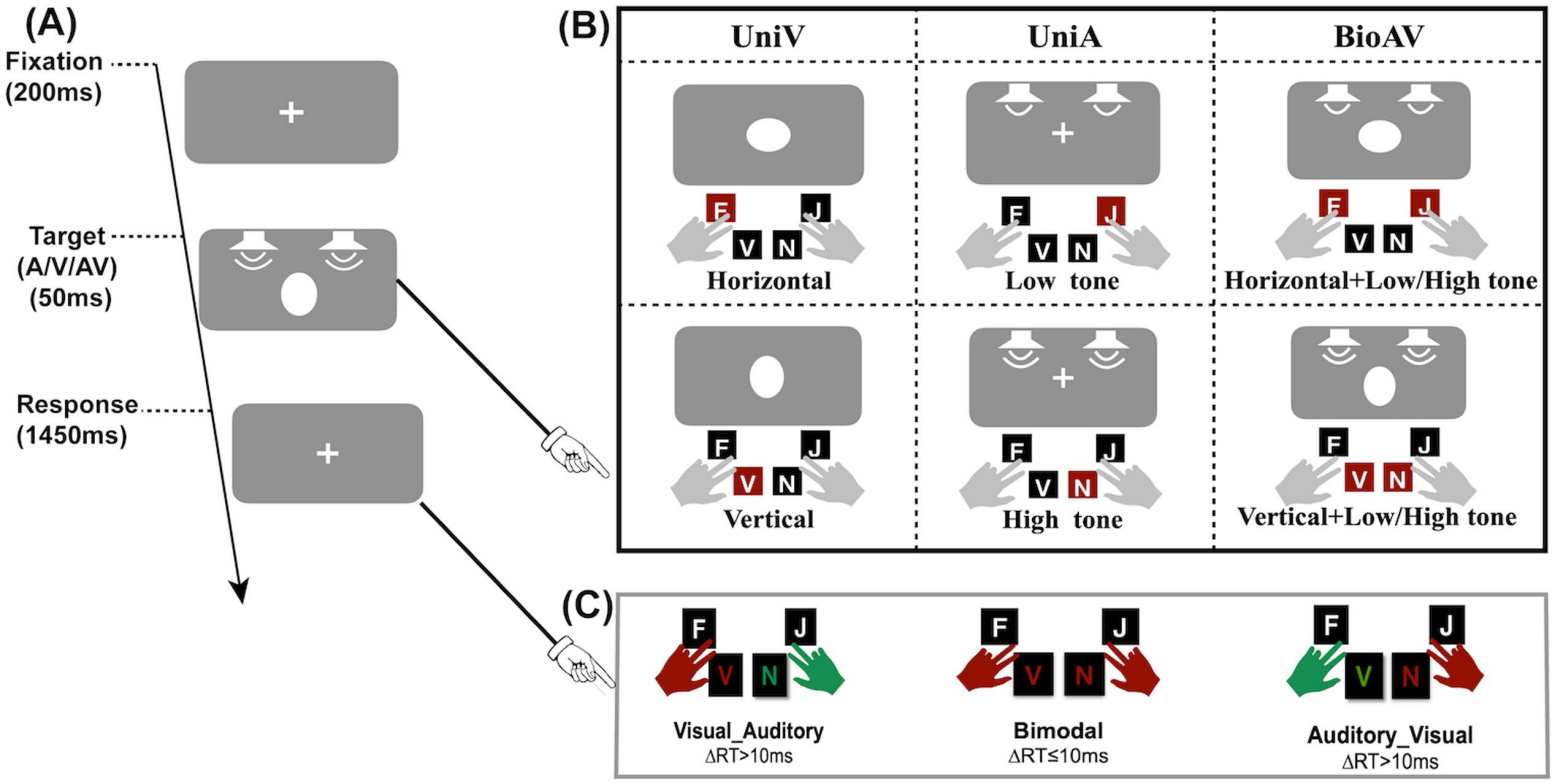
Experimental procedure and target type. **(A)** Schematic of the stimulus procedure. **(B)** Three stimulus types (unimodal visual, unimodal auditory, bimodal audiovisual). **(C)** Three button types for audiovisual stimuli: the red hand for pressing first and the green hand for pressing second. When the time between two hand presses was less than or equal to 10 ms, simultaneous key presses occurred.

The experimental material consisted of three stimulus types: auditory stimuli (A), visual stimuli (V), and bimodal audiovisual stimuli (AV). These are shown in Figure 1B. The V stimuli consisted of a white vertical ellipse and a white horizontal ellipse. The ellipses were formed by a 10% modification of the length of the horizontal and vertical diameters of the circle with a radius of 1.5° of view. The A stimuli consisted of a pure bass tone at 714 Hz and a pure treble tone at 1400 Hz. The AV stimuli consisted of two combination types, either horizontally low and vertically high (i.e., the horizontal ellipse appeared in combination with a pure bass at 714 Hz, and the vertical ellipse appeared in combination with a pure treble at 1,400 Hz) or horizontally high and vertically low (i.e., the horizontal ellipse appeared in combination with a pure treble at 1,400 Hz, and the vertical ellipse appeared in combination with a pure bass at 714 Hz).

The participants were located 60 cm from the computer screen and completed the experiment on a dimly lit, soundproof background. The visual stimuli were presented on a model 242EGSJ/93 LCD monitor with a gray background (RGB: 128, 128, 128), a visual size of 23.8 inches, a screen resolution of 1,920 × 1,080 pixels, and a refresh rate of 100 Hz. The auditory stimuli were two-channel pure tones at 4,000 Hz (5 ms in elevation and 5 ms out of elevation) presented by speakers (Brand: EDIFIER) placed behind the monitor and on both sides of the screen.

The experimental procedure was as follows (Figure 1A): first, a visual fixation stimulus was presented for 200 ms. Subsequently, unimodal A, unimodal V, or bimodal AV stimuli were presented randomly at the fixation location for 50 ms. Finally, a visual fixation stimulus was presented for 1,450 ms to allow participants to respond. Participants were prompted to respond to A stimuli and V stimuli by pressing four buttons on the keyboard. Specifically, the visual horizontal ellipse, visual vertical ellipse, auditory bass and auditory treble corresponded to the keys “F,” “V,” “J,” and “N,” respectively. Additionally, participants were asked to press two buttons at the same time if possible when audiovisual stimuli were presented simultaneously. Three button types for audiovisual stimuli are shown in Figure 1C. The three button types include priority response to visual stimuli, priority response to auditory stimuli, and simultaneous response to audiovisual stimuli. Participants pressed the answer key with two fingers of each hand (balanced between participants) (e.g., left middle finger for “F,” right middle finger for “J,” etc.) (see Figure 1).

This experiment used a two-factor within-subjects design, including unimodal target probability (10% V:40% A, 25% V:25% A, 40% V:10% A) and stimulus modality (V, A, AV). For the 10% V:40% A condition, each block contained 12 V trials and 48 A trials. For the 25% V:25% A condition, each block contained 30 V trials and 30 A trials. For the 40% V:10% A condition, each block contained 48 V trials and 12 A trials. The experiment consisted of a total of 9 blocks of 1,080 trials. Each block contained 120 trials, whereas there were 60 trials of the AV stimuli. Each unimodal target probability condition has 3 blocks. The total experiment time was approximately 2 h.

### 2.3 Data recording and analysis

#### 2.3.1 Behavioral analysis

To test the suitability of the sample size, we performed a sensitivity analysis of within-subjects repeated measures in the G*power toolbox (Faul et al., 2007). Input parameters: The parameter effect size *f* = 0.25, *α* err prob. = 0.05, and power (1–*β* err prob) = 0.80. Presentation 0.71 software (Neurobehavioral Systems, Inc.) was used for programming, stimuli presentation and response proportion and reaction time recording. For each participant, the study calculated the proportion of incorrect responses and the reaction time difference (∆RT) of correct responses for bimodal audiovisual stimuli. The calculation of the size of the visual dominance effect of Colavita in previous studies was mainly by comparing the proportion of incorrect responses for bimodal audiovisual stimuli (Colavita, 1974; Colavita et al., 1976). Incorrect responses include visual-only responses and auditory-only responses for bimodal audiovisual stimuli. For visual-only responses, participants responded to the visual stimuli only; for auditory-only responses, participants responded to the auditory stimuli only. Correct responses include V_A responses and A_V responses. V_A responses, in which participants first responded to the visual stimuli and then to the auditory stimuli; A_V responses, in which participants first responded to the auditory stimuli and then to the visual stimuli. The formulas used to calculate the magnitude of sensory dominance (∆RT difference) are as follows: In V_A responses, ∆RT_1_ = RT (auditory response)-RT (visual response); in A_V responses, ∆RT_2_ = RT (visual response)-RT (auditory response). ∆RT_1_ or ∆RT_2_ ≤ 10 ms indicates simultaneous responses (Fang et al., 2020; Wang et al., 2021).

Specifically, first, the study analyzed a 3 unimodal target probability (10% V:40% A, 25% V:25, 40% V:10% A) × 2 type of incorrect bimodal trials (visual-only vs. auditory-only) repeated-measures analysis of variance (ANOVA) to verify the existence of the Colavita effect by observing whether the main effect of type of incorrect bimodal trials was significant (Colavita, 1974). Second, the study analyzed the ∆RT, in which the participants pressed two keys at different times and conducted 3 unimodal target probability (10% V:40% A, 25% V: 25% A, 40% V:10% A) × 2 types of correctly responded bimodal trials (V_A vs. A_V) repeated-measures ANOVA to verify the existence of the Colavita effect (Fang et al., 2020; Wang et al., 2021).

#### 2.3.2 ERPs analysis

The ERP data were recorded by a Brain Product workstation (Germany) using a 32-lead actiCHamp electrode cap expanded according to the International Ag/AgCl Electrode 10–20 System with Brain Vision Recorder 2.0 software. A BrainAmp DC amplifier was used (low pass 30 Hz; high pass 0.01 Hz; slope = 24 dB/octave; sampling frequency 500 Hz). The left ear was used as the reference electrode, the forehead was grounded, and electrodes were placed approximately 1.5 cm above and below the left eye to record vertical electrooculograms (vEOGs) and approximately 1.5 cm lateral to the left eye to record horizontal electrooculograms (hEOGs). Throughout the task, electrode impedances were maintained below 5 kΩ. Digital filtering with a bandpass of 0.1–30 Hz was used to process the EEG data.

The EEG data were segmented for 1,000 ms starting 200 ms before stimulus onset. The waveforms were baseline corrected according to the 200 ms period before stimulation. Trials with EEG voltages exceeding ±80 μV were discarded before averaging. Correct response trials were used to calculate mean event-related potentials. EEGLAB (14.0) with MATLAB (MATLAB and Statistics Toolbox Release 2018b, The MathWorks, Inc., Natick, Massachusetts, USA.) software was used for preprocessing.

On the basis of the analysis results and the mean latency of N1/P2/N2, we selected three time windows (N1: 150–170 ms; P2: 200–250 ms; N2: 290–330 ms). In these time windows, electrodes were selected for which ERPs in the V_A type differed from ERPs in the A_V type (N1: F3, Fz, F4; P2: Cz, Pz, C3/4, P3/4; and N2: F3, Fz, F4). We focused on those ERP components and electrodes considering that previous studies have related them either to expectation (Melloni et al., 2011; Stojanoski and Niemeier, 2015). The average amplitude of each selected electrode was calculated for the selected time window. In each time window, the mean amplitude data were analyzed using repeated-measures ANOVA with factors of correctly responded bimodal trials (V_A/A_V) and unimodal target probability (10% V:40% A, 25% V:25% A, 40% V:10% A). The Greenhouse–Geisser epsilon or Bonferroni correction was used for non-sphericity or *post hoc* comparisons. The statistical level was set at 0.05. The effect sizes of Cohen’s *d* or partial eta-squared ( $\eta_{p}^{2}$ ) were calculated for mean comparisons or ANOVA, respectively.

## 3 Results

### 3.1 Behavioral data

#### 3.1.1 Proportion of different types of bimodal trials

**Figure 2:**
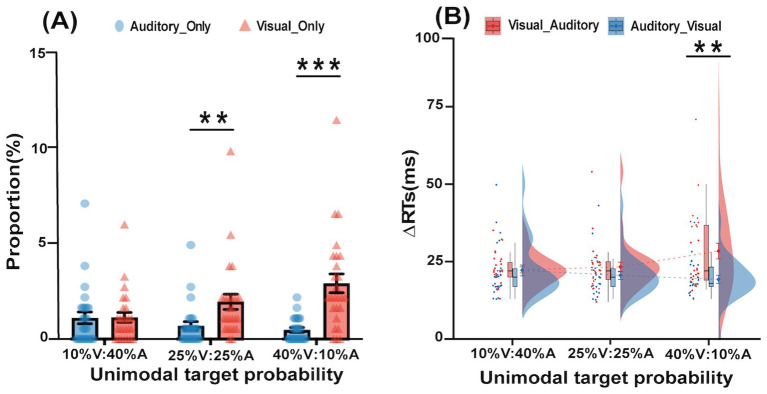
Proportion and ∆RT in the bimodal trials of the experiment. **(A)** Differences between the proportions of two types of incorrect bimodal trials (Visual_Only, Auditory_Only) in three different unimodal target probability (10% V:40% A, 25% V:25% A, 40% V:10% A) conditions. **(B)** Differences between the ∆RTs of two types of correctly responded bimodal trials (Visual_Auditory, Auditory_Visual) in three different unimodal target probabilities (10% V:40% A, 25% V:25% A, 40% V:10% A) conditions (*p < 0.05, **p < 0.01,***p < 0.001). The error bars indicate standard errors.

The proportions of incorrect bimodal trials (i.e., visual_only and auditory_only bimodal trials) were submitted to a 3 unimodal target probability (10% V:40% A, 25% V:25% A, 40% V:10% A) × 2 types of incorrect bimodal trials (visual_only vs. auditory_only) repeated-measures ANOVA; this is shown in Figure 2A. The main effect of the type of incorrect bimodal trial was significant *F*(1,25) = 23.45, *p* < 0.001, $\eta_{p}^{2}$ = 0.48, indicating that the proportion of visual_only trials (2.0%) was significantly larger than that of auditory_only trials (0.7%), suggesting that there was the Colavita effect. The main effect of the unimodal target probability was significant *F*(1.62,40.56) = 5.16, *p* = 0.02, $\eta_{p}^{2}$ = 0.17, indicating that the proportion of 40% V:10% A unimodal target (1.7%) was significantly larger than the 10% V:40% A unimodal target (1.1%). In addition, the interaction effect between the unimodal target probability and the type of error was significant, *F*(1.39,34.78) = 9.12, *p* = 0.002, $\eta_{p}^{2}$ = 0.27. The simple effect analysis demonstrated that the proportion of visual_only trials was significantly larger than the auditory_only trials in the 25% V:25% A and 40% V:10% A conditions (all *p* < 0.01).

#### 3.1.2 Reaction times of different types of bimodal trials

For RTs in the bimodal trials, a 3 unimodal target probability (10% V:40% A, 25% V:25% A, 40% V:10% A) × 2∆RT of correctly responded bimodal trials (V_A vs. A_V) repeated-measures ANOVA was performed, as shown in Figure 2B. The main effect of the unimodal target probability was not significant, *F*(2,50) = 1.25, *p* = 0.30, $\eta_{p}^{2}$ = 0.05. The main effect of the ∆RT of correctly responded bimodal trials was significant, *F*(1,25) = 9.64, *p* = 0.005, $\eta_{p}^{2}$ = 0.28, indicating that the ∆RT of the V_A type (24.69 ms) was significantly higher than the ∆RT of the A_V type (20.67 ms), suggesting that there was Colavita effect. The interaction was also significant, *F*(2,50) = 5.54, *p =* 0.007, $\eta_{p}^{2}$ = 0.18. Further tests of simple effects revealed that in the 40% V:10% A condition, the ∆RT of the V_A type (28.42 ms) was larger than the ∆RT of the A_V type (19.27 ms), *p* = 0.002.

### 3.2 ERP data

#### 3.2.1 N1 (150–170 ms)

**Figure 3:**
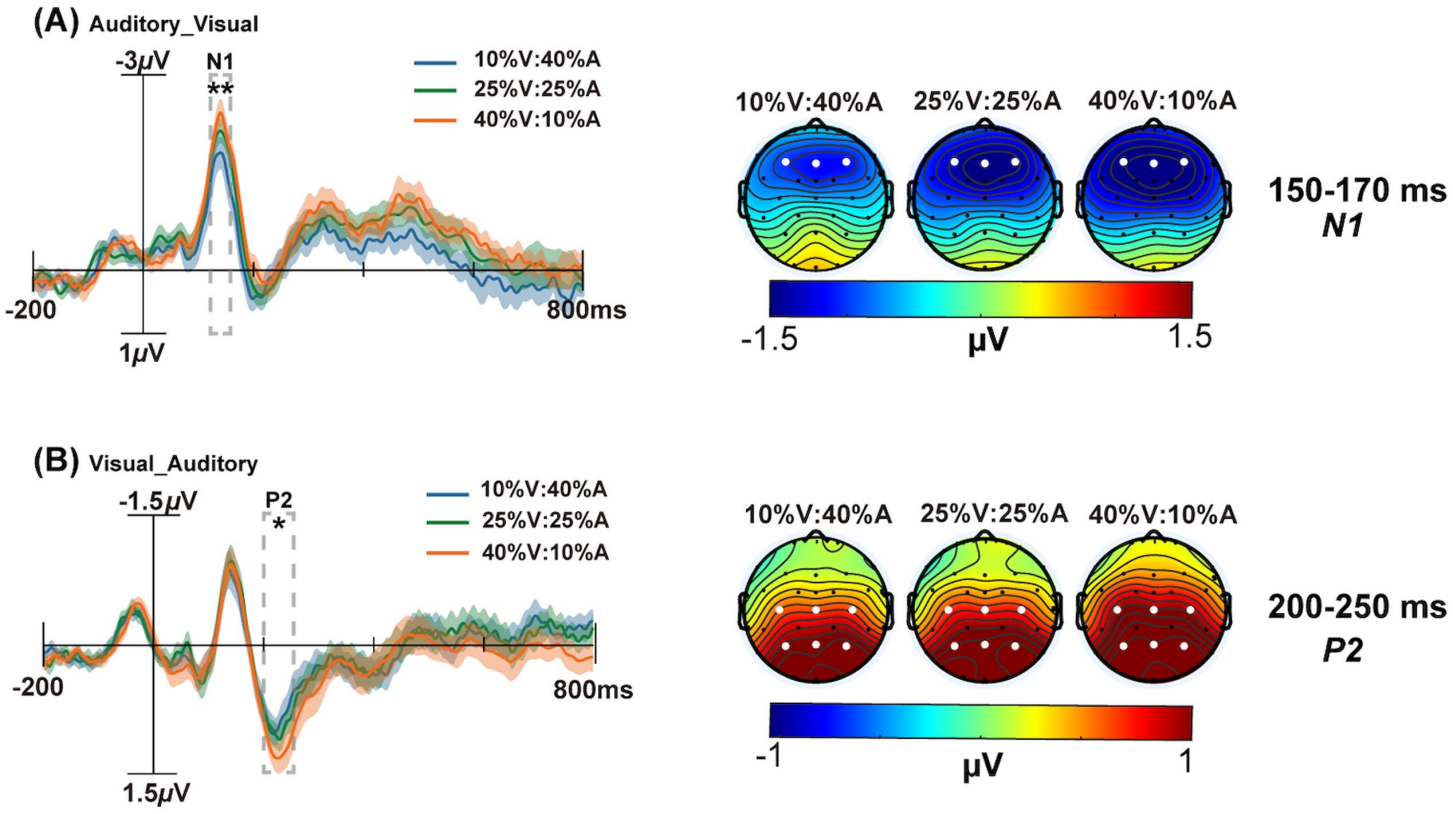
The grand average ERPs of the maximum difference between the 10% V:40% A (blue solid line) and 40% V:10% A (yellow solid line) conditions are shown on the left. The scalp topographies at 10% V:40% A, 25% V:25% A and 40% V:10% A are shown on the right. **(A)** The analysis time window for N1 (150–170 ms) is shaded gray on the F3, Fz, and F4 electrodes in the Auditory_Visual condition. **(B)** The analysis time window for P2 (200–250 ms) is shaded gray on the C3, Cz, C4, P3, Pz, and P4 electrodes in the Visual_Auditory condition. The shadows indicate the SEs of ERPs. *p < 0.05, **p < 0.01.

For the N1 component, a 3 unimodal target probability (10% V:40% A, 25% V:25% A, 40% V:10% A) × 2 type of correctly responded bimodal trials (V_A vs. A_V) repeated-measures ANOVA was performed, as shown in Figure 3A. The main effect of the unimodal target probability was significant, *F*(2,50) = 4.88, *p* = 0.01, $\eta_{p}^{2}$ = 0.16. The main effect of correctly responded bimodal trials was not significant, *F*(1,25) = 1.32, *p* = 0.26, $\eta_{p}^{2}$ = 0.05. The interaction was significant, *F*(2,50) = 3.41, *p* = 0.04, $\eta_{p}^{2}$ = 0.12. Further tests of simple effects revealed that the 40% V:10% A condition (−1.59 μV) had significantly more negative effects than the 10% V:40% A condition (−1.23 μV) under the A_V type (*p* = 0.005).

#### 3.2.2 P2 (200–250 ms)

**Figure 4:**
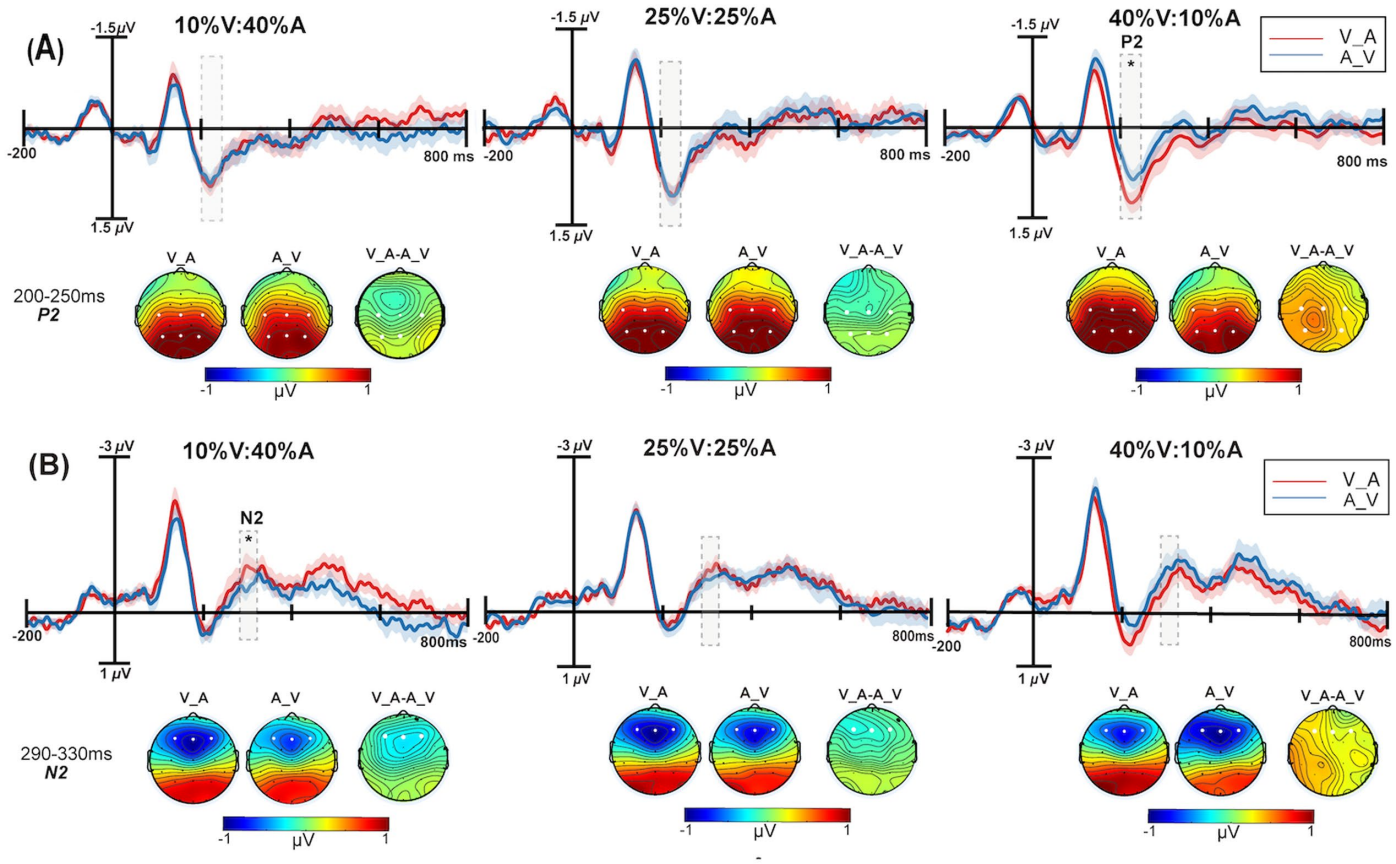
The grand average ERPs with the greatest differences between the Auditory_Visual (blue solid line) and Visual_Auditory (red solid line) conditions are shown. The scalp topographies of Auditory_Visual, Visual_Auditory and the differential wave topographic map of Visual_Auditory minus the Auditory_Visual are shown. **(A)** The analysis time window for P2 (200–250 ms) is shaded gray for the C3, Cz, C4, P3, Pz, and P4 electrodes at 10% V:40% A, 25% V:25% A, and 40% V:10% A. **(B)** The analysis time window for N2 (290–330 ms) is shaded gray on the F3, Fz, and F4 electrodes at 10% V:40% A, 25% V:25% A, and 40% V:10% A. The shadows indicate the SEs of ERPs. *p < 0.05.

For the P2 component, a 3 unimodal target probability (10% V:40% A, 25% V:25% A, 40% V:10% A) × 2 type of correctly responded bimodal trials (V_Avs. A_V) repeated-measures ANOVA was performed. The main effect of the unimodal target probability was not significant, *F*(2,50) = 1.52, *p* = 0.22, $\eta_{p}^{2}$ = 0.06. The main effect of correctly responded bimodal trials was not significant, *F*(1,25) = 3.87, *p* = 0.06, $\eta_{p}^{2}$ = 0.13. The interaction was significant, *F*(2,50) = 3.36, *p* = 0.05, $\eta_{p}^{2}$ = 0.12. Further tests of simple effects revealed that the 40% V:10% A condition (0.99 μV) had more positive effects than did the 10% V:40% A condition (0.84 μV) under the V_A type (*p =* 0.06) (Figure 3B). The P2 amplitude of V_A (1.01 μV) was significantly more positive than that of A_V (0.86 μV) under the 40% V:10% A condition (*p* = 0.01) (Figure 4A).

#### 3.2.3 N2 (290–330 ms)

For the N2 component, a 3 unimodal target probability (10% V:40% A, 25% V:25% A, 40% V:10% A) × 2 type of correctly responded bimodal trials (V_Avs. A_V) repeated-measures ANOVA was performed, this is shown in Figure 4B. The main effect of the unimodal target probability was not significant, *F*(2,50) = 0.56, *p* = 0.57, $\eta_{p}^{2}$ = 0.02. The main effect of correctly responded bimodal trials was not significant, *F*(1,25) = 0.65, *p* = 0.043, $\eta_{p}^{2}$ = 0.03. The interaction was significant, *F*(2,50) = 4.03, *p* = 0.02, $\eta_{p}^{2}$ = 0.14. Simple effect analysis revealed that the N2 amplitude of V_A was significantly more negative than that of A_V in the 10% V:40% A condition (*p* = 0.03).

## 4 Discussion

The present study aimed to investigate the modulatory effect of modality expectation on the Colavita visual dominance effect using ERPs measurements. First, the behavioral results revealed a significant Colavita visual dominance effect when the unimodal target probability was set to 25% V:25% A and 40% V:10% A, and the proportion of visual-only responses was significantly greater than the proportion of auditory-only responses in both conditions. The current results are partly consistent with those of previous studies that reported a significantly number of visual-only responses when the expectation was directed to unimodal visual stimulus (40% V:10% A condition) (Sinnett et al., 2007; Koppen and Spence, 2007b). In contrast, no significant difference was found between visual-only and auditory-only responses when the expectation was directed to unimodal auditory stimulus (10% V:40% A condition) (Colavita, 1974; Sinnett et al., 2007; Koppen and Spence, 2007b). Second, a larger ∆RT of V_A was found when the expectation was directed to visual modality (40% V:10% A condition) and when the top-down expectation was directed to auditory modality (10% V:40% A condition) leading to an equivalent ∆RT between V_A and A_V. Previous studies suggested that visual dominance might reflect the compensation mechanism because auditory stimuli are automatically alerted, whereas visual stimuli are not (Posner et al., 1976; Chen and Huang, 2021). In addition, in the present study, participants tended to actively direct their expectations toward visual stimuli, especially when the unimodal visual target probability was set to 40% V. Finally, visual stimuli of bimodal audiovisual stimulus is perceived faster than auditory stimuli is and eventually leads to a preferential response to visual stimuli.

For the ERPs data, the results revealed that the mean amplitude of the N1 component (150–170 ms) in the A_V type was larger in the 40% V:10% A condition than in the 10% V:40% A condition around the frontal region. Second, the mean amplitude of the P2 component (200–250 ms) in the 40% V:10% A condition was larger in the V_A than in the A_V type around the central-parietal region. The mean amplitude of the P2 component (200–250 ms) in the V_A type was larger in the 40% V:10% A condition than in the 10% V:40% A condition. Finally, the mean amplitude of the N2 component in the 10% V:40% A condition (290–330 ms) was larger in the V_A than in the A_V type in the frontal region.

To illustrate the effect of modality expectation on sensory dominance, we analyzed the time course of visual and auditory expectations in the A_V type. In line with our hypothesis, the ERP amplitude of visual modal expectation (40% V:10% A) was significantly greater than that of auditory modal expectation (10% V:40% A) in the N1 time window. Previous studies suggested that the N1 component was an exogenous and robust auditory ERP component (Teder et al., 1993; Luo and Wei, 1999; Tomé et al., 2015). Additionally, some other evidence indicates that the N1 component might be involved in the top-down attention switching mechanism (Sussman et al., 2003; Zanto et al., 2010; Bidet-Caulet et al., 2015). For example, one study used binaural hearing experiments to explore the physiological and psychological mechanisms of selective auditory attention in humans and suggested that the N1 component might be associated with attentionally allocating mechanisms, especially when participants must selectively pay attention to sounds in one ear and ignore sounds (Woldorff and Hillyard, 1991). This evidence indicates that selective auditory attention can influence the early stage of sensory input, thereby influencing the processing of auditory stimuli. Combined with our studies, the auditory N1 component was found only in the A_V type, indicating that expectations may influence the perception of auditory stimuli by endogenously manipulating attention at an early stage. Similarly, Grau et al. (2007) suggested that the N1 component was involved in the top-down mechanism of attention switching, which could trigger an attention-capturing signal for conscious perception of the stimulus (Grau et al., 2007). Therefore, combining Woldorff and Hillyardas with Grau’s opinions, the significantly greater N1 amplitude elicited by the 40% V:10% A condition in the present study might indicate a salient perception demand resulting from lower expectations of the auditory stimulus capturing exogenous attention.

Furthermore, we analyzed the time course of visual and auditory expectations in the V_A type. The ERP amplitude of visual modal expectation (40% V:10% A) was significantly larger than that of auditory modal expectation (10% V:40% A) in the P2 time window in the central-parietal area. The P2 component is thought to be possibly related to top-down expectation engagement (Federmeier and Kutas, 2002; Freunberger et al., 2007). One study revealed that the amplitude of the P2 component of the left hemisphere (right field of view) has a larger amplitude for expected visual pictures than for unexpected visual pictures from an unexpected category, which indicates that the left hemisphere can use top-down expectations to analyze visual features more effectively when processing contextual information (Federmeier and Kutas, 2002). In addition, the P2 component may also be associated with increased cognitive resource processing needs (Lai et al., 2020). Lai et al. (2020) reported that older adults presented greater P2 amplitudes when dealing with target object ambiguity, which may indicate that they need more cognitive resource processing to address perceived ambiguity. The above evidence indicated that the P2 component might be associated with cognitive resource modulation caused by top-down expectations, so visual stimuli may occupy more cognitive resources under conditions of visual modal expectation (40% V:10% A). In this study, as the unimodal visual probability increased, more visual stimuli required more cognitive processing; thus, a significantly greater ERP amplitude of P2 was found under the visual expectation (40% V:10% A) condition than under the auditory expectation (10% V:40% A) condition.

Additionally, it must be noted that some previous studies reported no significant difference between the amplitudes of V_A and A_V in the P2 time window (Huang et al., 2015). This result may reflect the unimodal probabilistic manipulation difference between the present study and the previous study, which maintained the proportions of visual, auditory, and audiovisual stimuli at 40%:40%:20%. In previous studies, there was no change in modality expectation for unimodal probabilistic manipulation. In contrast, in the present study, audiovisual stimuli were maintained at 50%, and three conditions were used to modulate modality expectations: 10% V:40% A, 25% V:25% A, and 40% V:10% A. Therefore, it may be that the adjustment of modality expectation revealed a significant difference between V_A and A_V in the P2 time window. In addition, Spence et al. (2012) proposed a biased competition hypothesis stating that sensory systems essentially compete with each other (Duncan et al., 1997; Spence et al., 2012). When visual signals tend to dominate, neural activity for auditory signals is suppressed, eventually manifesting as a visual dominance effect during the response phase. Therefore, the ERP amplitude of P2 in the V_A type was larger than that in the A_V type, indicating the Colavita visual dominance effect.

Importantly, partly consistent with previous findings (Barceló et al., 2000; Folstein and Van Petten, 2008; Grossheinrich et al., 2013), this study also revealed an N2 component in the prefrontal region in the 10% V:40% A condition. The mean amplitude of the V_A type was significantly larger than in the A_V type. Previous studies have shown that the N2 component is an auditory ERP component (Folstein and Van Petten, 2008). Alternatively, some researchers have indicated that the N2 component may be associated with target stimulus probabilities (Bruin and Wijers, 2002). Low-probability stimuli induce larger negative wave peaks in the N2 time window (Bruin and Wijers, 2002). In the present study, the same auditory N2 was present, and N2 may be associated with a low probability of stimulation such that fewer visual stimulus conditions evoked greater negative wave peaks in the V_A type. More importantly, some studies have suggested that the N2 component might be associated with conflict resolution (Nieuwenhuis et al., 2003; Yeung et al., 2004). For example, Yeung et al. (2004) used the flanker task to investigate the electrophysiology mechanism of conflict monitoring on correct trials by manipulating the conflict level. N2 appeared in the attempts to resolve the response conflict, and the mean N2 amplitude was greater in the incongruent trials than in the congruent trials. Combining the above evidence with our results, the N2 component in this study may also be associated with conflicting responses, which occur when the visual precedence response (V_A) occurs under auditory modal expectation (10% V:40% A) condition. In the present study, top-down attention might be endogenously biased to the auditory modality when the expectation was directed to the auditory modality, whereas visual stimuli were preferentially responded to, ultimately producing response conflict and the emergence of the N2 component.

Overall, the current study investigated the electrophysiological mechanisms of Colavita visual dominance under different modality expectations by adjusting the unimodal target probability. First, the behavioral results of this study are partly consistent with some previous studies which suggested that Colavita visual dominance effect could be modulated by modality-specific expectation (Sinnett et al., 2007; Wang et al., 2023). Importantly, under the condition of visual expectation, we found that the mean amplitude of V_A regarding the P2 component was significantly greater than that of A_V. Under auditory expectation conditions, we found that the average amplitude of V_A in the N2 component was significantly greater than that of A_V. According to the electroencephalogram (EEG) results, when visual signals tend to dominate, the neural activity of auditory signals is suppressed, which ultimately manifests as the Colavita visual dominance effect and the emergence of the P2 component. When expectations are directed toward the auditory modality, top-down attention may endogenously bias toward the auditory modality, while visual stimuli are preferentially responded to, ultimately resulting in response conflict and the emergence of the N2 component. We further identified distinct ERP components under different modality expectation conditions, which demonstrated that modality expectation modulates the Colavita visual dominance effect at later stages. This study provides a new perspective for understanding at which specific stages different factors influence sensory dominance. In addition, the underlying neural mechanisms by which modality-specific expectations modulate sensory dominance can be further explored in future neuroimaging or neurophysiological studies.

## 5 Conclusion

Using the high temporal resolution of event-related potentials (ERPs), we investigated how modality expectation modulates sensory dominance by manipulating the different unimodal target probabilities used in previous related Colavita studies. The behavioral results revealed a significantly larger Colavita visual dominance effect when modality expectation was directed to visual sensory stimulus (i.e., 40% V:10% A condition). Further ERPs results revealed that the N1 component (150–170 ms) in the A_V type was larger in the 40% V:10% A condition than in the 10% V:40% A condition in the frontal region. The mean amplitude of the P2 component (200–250 ms) in the 40% V:10% A condition was larger in the V_A type than in the A_V type in the central-parietal region. The mean amplitude of the P2 component (200–250 ms) in the V_A type was larger in the 40% V:10% A condition than in the 10% V:40% A condition. The mean amplitude of the N2 component in the 10% V:40% A condition (290–330 ms) was larger in the V_A type than in the A_V type in the frontal region. In conclusion, these results tentatively indicate that increasing the unimodal target probability may lead to greater top-down expectation of the target modality, and then sensory dominance emerges in the late phase in response to bimodal audiovisual stimuli.

## Data Availability

The raw data supporting the conclusions of this article will be made available by the authors, without undue reservation.

## References

[ref1] Barceló F. Suwazono S. Knight R. T. (2000). Prefrontal modulation of visual processing in humans. Nat. Neurosci. 3, 399–403. doi: 10.1038/73975, PMID: 10725931

[ref2] Bidet-Caulet A. Bottemanne L. Fonteneau C. Giard M. H. Bertrand O. (2015). Brain dynamics of distractibility: interaction between top-down and bottom-up mechanisms of auditory attention. Brain Topogr. 28, 423–436. doi: 10.1007/s10548-014-0354-x, PMID: 24531985

[ref3] Bruin K. J. Wijers A. A. (2002). Inhibition, response mode, and stimulus probability: a comparative event-related potential study. Clin. Neurophysiol. 113, 1172–1182. doi: 10.1016/s1388-2457(02)00141-4, PMID: 12088714

[ref4] Chen Y. C. Huang P. C. (2021). Stimulus temporal uncertainty balances intersensory dominance. Psychon. Bull. Rev. 28, 1874–1884. doi: 10.3758/s13423-021-01959-0, PMID: 34159527

[ref5] Colavita F. B. (1974). Human sensory dominance. Percept. Psychophys. 16, 409–412. doi: 10.3758/BF03203962

[ref6] Colavita F. Tomko R. Weisberg D. (1976). Visual prepotency and eye orientation. Bull. Psychon. Soc. 8, 25–26. doi: 10.3758/BF03337062

[ref7] Desimone R. Duncan J. (1995). Neural mechanisms of selective visual attention. Annu. Rev. Neurosci. 18, 193–222. doi: 10.1146/annurev.ne.18.030195.001205 7605061

[ref8] Dugué L. Merriam E. P. Heeger D. J. Carrasco M. (2020). Differential impact of endogenous and exogenous attention on activity in human visual cortex. Sci. Rep. 10:21274. doi: 10.1038/s41598-020-78172-x, PMID: 33277552 PMC7718281

[ref9] Duncan J. Humphreys G. Ward R. (1997). Competitive brain activity in visual attention. Curr. Opin. Neurobiol. 7, 255–261. doi: 10.1016/s0959-4388(97)80014-1, PMID: 9142748

[ref10] Egeth H. Sager L. (1977). On the locus of visual dominance. Percept. Psychophys. 22, 77–86. doi: 10.3758/BF03206083

[ref11] Fang Y. Li Y. Xu X. Tao H. Chen Q. (2020). Top-down attention modulates the direction and magnitude of sensory dominance. Exp. Brain Res. 238, 587–600. doi: 10.1007/s00221-020-05737-7, PMID: 31996936

[ref12] Faul F. Erdfelder E. Lang A. G. Buchner A. (2007). G*power 3: a flexible statistical power analysis program for the social, behavioral, and biomedical sciences. Behav. Res. Methods 39, 175–191. doi: 10.3758/bf03193146, PMID: 17695343

[ref13] Federmeier K. D. Kutas M. (2002). Picture the difference: electrophysiological investigations of picture processing in the two cerebral hemispheres. Neuropsychologia 40, 730–747. doi: 10.1016/s0028-3932(01)00193-2, PMID: 11900725

[ref14] Folstein J. R. Van Petten C. (2008). Influence of cognitive control and mismatch on the N2 component of the ERP: a review. Psychophysiology 45, 152–170. doi: 10.1111/j.1469-8986.2007.00602.x, PMID: 17850238 PMC2365910

[ref15] Freunberger R. Klimesch W. Doppelmayr M. Höller Y. (2007). Visual P2 component is related to theta phase-locking. Neurosci. Lett. 426, 181–186. doi: 10.1016/j.neulet.2007.08.062, PMID: 17904744

[ref16] Ghazanfar A. A. Lemus L. (2010). Multisensory integration: vision boosts information through suppression in auditory cortex. Curr. Biol. 20, R22–R23. doi: 10.1016/j.cub.2009.11.046, PMID: 20152139

[ref17] Grau C. Fuentemilla L. Marco-Pallarés J. (2007). Functional neural dynamics underlying auditory event-related N1 and N1 suppression response. NeuroImage 36, 522–531. doi: 10.1016/j.neuroimage.2007.03.027, PMID: 17499521

[ref18] Grossheinrich N. Reinl M. Pogarell O. Karch S. Mulert C. Brueckl M.. (2013). Effects of low frequency prefrontal repetitive transcranial magnetic stimulation on the N2 amplitude in a GoNogo task. PLoS One 8:e67136. doi: 10.1371/journal.pone.0067136, PMID: 23826214 PMC3694966

[ref19] Hartcher-O'Brien J. Gallace A. Krings B. Koppen C. Spence C. (2008). When vision 'extinguishes' touch in neurologically-normal people: extending the Colavita visual dominance effect. Exp. Brain Res. 186, 643–658. doi: 10.1007/s00221-008-1272-5, PMID: 18301885

[ref20] Hecht D. Reiner M. (2009). Sensory dominance in combinations of audio, visual and haptic stimuli. Exp. Brain Res. 193, 307–314. doi: 10.1007/s00221-008-1626-z, PMID: 18985327

[ref21] Huang S. Li Y. Zhang W. Zhang B. Liu X. Mo L.. (2015). Multisensory competition is modulated by sensory pathway interactions with Fronto-sensorimotor and default-mode network regions. J. Neurosci. 35, 9064–9077. doi: 10.1523/JNEUROSCI.3760-14.2015, PMID: 26085631 PMC6605163

[ref22] Hutmacher F. (2019). Why is there so much more research on vision than on any other sensory modality? Front. Psychol. 10:2246. doi: 10.3389/fpsyg.2019.02246, PMID: 31636589 PMC6787282

[ref23] Kok P. Rahnev D. Jehee J. F. Lau H. C. de Lange F. P. (2012). Attention reverses the effect of prediction in silencing sensory signals. Cereb. Cortex 22, 2197–2206. doi: 10.1093/cercor/bhr310, PMID: 22047964

[ref24] Koppen C. Levitan C. A. Spence C. (2009). A signal detection study of the Colavita visual dominance effect. Exp. Brain Res. 196, 353–360. doi: 10.1007/s00221-009-1853-y, PMID: 19488743

[ref25] Koppen C. Spence C. (2007a). Assessing the role of stimulus probability on the Colavita visual dominance effect. Neurosci. Lett. 418, 266–271. doi: 10.1016/j.neulet.2007.03.032, PMID: 17398003

[ref26] Koppen C. Spence C. (2007b). Seeing the light: exploring the Colavita visual dominance effect. Exp. Brain Res. 180, 737–754. doi: 10.1007/s00221-007-0894-3, PMID: 17333012

[ref27] Lai L. Y. Frömer R. Festa E. K. Heindel W. C. (2020). Age-related changes in the neural dynamics of bottom-up and top-down processing during visual object recognition: an electrophysiological investigation. Neurobiol. Aging 94, 38–49. doi: 10.1016/j.neurobiolaging.2020.05.010, PMID: 32562874

[ref28] Langner R. Kellermann T. Boers F. Sturm W. Willmes K. Eickhoff S. B. (2011). Modality-specific perceptual expectations selectively modulate baseline activity in auditory, somatosensory, and visual cortices. Cereb. Cortex 21, 2850–2862. doi: 10.1093/cercor/bhr083, PMID: 21527785

[ref29] Lewkowicz D. J. Ghazanfar A. A. (2009). The emergence of multisensory systems through perceptual narrowing. Trends Cogn. Sci. 13, 470–478. doi: 10.1016/j.tics.2009.08.004, PMID: 19748305

[ref30] Li Y. Liu M. Zhang W. Huang S. Zhang B. Liu X.. (2017). Neurophysiological correlates of visual dominance: a lateralized readiness potential investigation. Front. Psychol. 8:303. doi: 10.3389/fpsyg.2017.00303, PMID: 28303113 PMC5332361

[ref31] Luo Y. J. Wei J. H. (1999). Cross-modal selective attention to visual and auditory stimuli modulates endogenous ERP components. Brain Res. 842, 30–38. doi: 10.1016/s0006-8993(99)01799-0, PMID: 10526092

[ref32] Manns M. Güntürkün O. (2009). Dual coding of visual asymmetries in the pigeon brain: the interaction of bottom-up and top-down systems. Exp. Brain Res. 199, 323–332. doi: 10.1007/s00221-009-1702-z, PMID: 19153723

[ref33] Melloni L. Schwiedrzik C. M. Müller N. Rodriguez E. Singer W. (2011). Expectations change the signatures and timing of electrophysiological correlates of perceptual awareness. J. Neurosci. 31, 1386–1396. doi: 10.1523/jneurosci.4570-10.2011, PMID: 21273423 PMC6623627

[ref34] Murray M. M. Eardley A. F. Edginton T. Oyekan R. Smyth E. Matusz P. J. (2018). Sensory dominance and multisensory integration as screening tools in aging. Sci. Rep. 8:8901. doi: 10.1038/s41598-018-27288-2, PMID: 29891964 PMC5995929

[ref35] Nieuwenhuis S. Yeung N. van den Wildenberg W. Ridderinkhof K. R. (2003). Electrophysiological correlates of anterior cingulate function in a go/no-go task: effects of response conflict and trial type frequency. Cogn. Affect. Behav. Neurosci. 3, 17–26. doi: 10.3758/cabn.3.1.17 12822595

[ref36] Posner M. I. Nissen M. J. Klein R. M. (1976). Visual dominance: an information-processing account of its origins and significance. Psychol. Rev. 83, 157–171. doi: 10.1037/0033-295x.83.2.157 769017

[ref37] Rungratsameetaweemana N. Serences J. T. (2019). Dissociating the impact of attention and expectation on early sensory processing. Curr. Opin. Psychol. 29, 181–186. doi: 10.1016/j.copsyc.2019.03.014, PMID: 31022561 PMC6756985

[ref38] Sinnett S. Spence C. Soto-Faraco S. (2007). Visual dominance and attention: the Colavita effect revisited. Percept. Psychophys. 69, 673–686. doi: 10.3758/bf03193770, PMID: 17929691

[ref39] Spence C. Parise C. Chen Y. C. (2012). “The Colavita visual dominance effect” in the neural bases of multisensory processes. eds. Murray M. M. Wallace M. T. (Boca Raton, FL: CRC Press/Taylor & Francis). 22593876

[ref40] Stein B. E. Stanford T. R. (2008). Multisensory integration: current issues from the perspective of the single neuron. Nat. Rev. Neurosci. 9, 255–266. doi: 10.1038/nrn2331, PMID: 18354398

[ref41] Stekelenburg J. J. Keetels M. (2016). The effect of synesthetic associations between the visual and auditory modalities on the Colavita effect. Exp. Brain Res. 234, 1209–1219. doi: 10.1007/s00221-015-4363-0, PMID: 26126803 PMC4828489

[ref42] Stojanoski B. Niemeier M. (2011). The timing of feature-based attentional effects during object perception. Neuropsychologia 49, 3406–3418. doi: 10.1016/j.neuropsychologia.2011.08.017, PMID: 21889519

[ref43] Stojanoski B. B. Niemeier M. (2014). Late electrophysiological modulations of feature-based attention to object shapes. Psychophysiology 51, 298–308. doi: 10.1111/psyp.12174, PMID: 24423181

[ref44] Stojanoski B. B. Niemeier M. (2015). Colour expectations during object perception are associated with early and late modulations of electrophysiological activity. Exp. Brain Res. 233, 2925–2934. doi: 10.1007/s00221-015-4362-1, PMID: 26139090

[ref45] Summerfield C. de Lange F. P. (2014). Expectation in perceptual decision making: neural and computational mechanisms. Nat. Rev. Neurosci. 15, 745–756. doi: 10.1038/nrn3838, PMID: 25315388

[ref46] Summerfield C. Egner T. (2009). Expectation (and attention) in visual cognition. Trends Cogn. Sci. 13, 403–409. doi: 10.1016/j.tics.2009.06.003, PMID: 19716752

[ref47] Sussman E. Winkler I. Schröger E. (2003). Top-down control over involuntary attention switching in the auditory modality. Psychon. Bull. Rev. 10, 630–637. doi: 10.3758/bf03196525, PMID: 14620357

[ref48] Talsma D. Senkowski D. Soto-Faraco S. Woldorff M. G. (2010). The multifaceted interplay between attention and multisensory integration. Trends Cogn. Sci. 14, 400–410. doi: 10.1016/j.tics.2010.06.008, PMID: 20675182 PMC3306770

[ref49] Teder W. Alho K. Reinikainen K. Näätänen R. (1993). Interstimulus interval and the selective-attention effect on auditory ERPs: “N1 enhancement” versus processing negativity. Psychophysiology 30, 71–81. doi: 10.1111/j.1469-8986.1993.tb03206.x, PMID: 8416064

[ref50] Tomé D. Barbosa F. Nowak K. Marques-Teixeira J. (2015). The development of the N1 and N2 components in auditory oddball paradigms: a systematic review with narrative analysis and suggested normative values. J. Neural Transm. 122, 375–391. doi: 10.1007/s00702-014-1258-3, PMID: 24961573

[ref51] Wang X. Wu Y. Xing Z. Cui X. Gao M. Tang X. (2023). Modal-based attention modulates the redundant-signals effect: role of unimodal target probability. Perception 52, 97–115. doi: 10.1177/03010066221136675, PMID: 36415087

[ref52] Wang A. Zhou H. Hu Y. Wu Q. Zhang T. Tang X.. (2021). Endogenous spatial attention modulates the magnitude of the Colavita visual dominance effect. Iperception 12:20416695211027186. doi: 10.1177/20416695211027186, PMID: 34290850 PMC8278468

[ref53] Witten I. B. Knudsen E. I. (2005). Why seeing is believing: merging auditory and visual worlds. Neuron 48, 489–496. doi: 10.1016/j.neuron.2005.10.020, PMID: 16269365

[ref54] Woldorff M. G. Hillyard S. A. (1991). Modulation of early auditory processing during selective listening to rapidly presented tones. Electroencephalogr. Clin. Neurophysiol. 79, 170–191. doi: 10.1016/0013-4694(91)90136-r, PMID: 1714809

[ref55] Xi Y. Lan Z. Chen Y. Zhang Q. Wu Z. Li G. (2023). Patients with epilepsy without cognitive impairment show altered brain networks in multiple frequency bands in an audiovisual integration task. Neurophysiol. Clin. 53:102888. doi: 10.1016/j.neucli.2023.102888, PMID: 37660635

[ref56] Yeung N. Botvinick M. M. Cohen J. D. (2004). The neural basis of error detection: conflict monitoring and the error-related negativity. Psychol. Rev. 111, 931–959. doi: 10.1037/0033-295X.111.4.931, PMID: 15482068

[ref57] Zampini M. Shore D. I. Spence C. (2005). Audiovisual prior entry. Neurosci. Lett. 381, 217–222. doi: 10.1016/j.neulet.2005.01.085, PMID: 15896473

[ref58] Zanto T. P. Rubens M. T. Bollinger J. Gazzaley A. (2010). Top-down modulation of visual feature processing: the role of the inferior frontal junction. NeuroImage 53, 736–745. doi: 10.1016/j.neuroimage.2010.06.012, PMID: 20600999 PMC2930130

[ref59] Zuanazzi A. Noppeney U. (2020). Modality-specific and multisensory mechanisms of spatial attention and expectation. J. Vis. 20:1. doi: 10.1167/jov.20.8.1, PMID: 32744617 PMC7438668

